# The centrosome protein NEDD1 as a potential pharmacological target to induce cell cycle arrest

**DOI:** 10.1186/1476-4598-8-10

**Published:** 2009-02-25

**Authors:** Vanessa Tillement, Laurence Haren, Nicolas Roullet, Chantal Etievant, Andreas Merdes

**Affiliations:** 1 Centre National de la Recherche Scientifique – Pierre Fabre, UMR 2587, 3 rue des Satellites, 31400 Toulouse, France

## Abstract

**Background:**

NEDD1 is a protein that binds to the gamma-tubulin ring complex, a multiprotein complex at the centrosome and at the mitotic spindle that mediates the nucleation of microtubules.

**Results:**

We show that NEDD1 is expressed at comparable levels in a variety of tumor-derived cell lines and untransformed cells. We demonstrate that silencing of NEDD1 expression by treatment with siRNA has differential effects on cells, depending on their status of p53 expression: p53-positive cells arrest in G1, whereas p53-negative cells arrest in mitosis with predominantly aberrant monopolar spindles. However, both p53-positive and -negative cells arrest in mitosis if treated with low doses of siRNA against NEDD1 combined with low doses of the inhibitor BI2536 against the mitotic kinase Plk1. Simultaneous reduction of NEDD1 levels and inhibition of Plk1 act in a synergistic manner, by potentiating the anti-mitotic activity of each treatment.

**Conclusion:**

We propose that NEDD1 may be a promising target for controlling cell proliferation, in particular if targeted in combination with Plk1 inhibitors.

## Background

The centrosome is a cellular structure responsible for the nucleation and organisation of microtubules. Because the centrosome is duplicated prior to cell division, during S-phase, the resulting two organising centres ensure the assembly of a bipolar spindle in mitosis, allowing the correct segregation of chromosomes. Considerable interest has focused on the role of the centrosome in cancer, because frequent abnormalities are found in tumor cells, such as supernumerary centrosomes or increased expression of centrosome proteins [1,2]. This phenomenon is also termed "centrosome amplification", and has often been correlated with aggressive tumor growth. Although it has not been formally demonstrated that centrosome amplification can cause cancer, centrosome abnormalities can generate defective mitotic spindles and therefore lead to mis-segregation of chromosomes and to aneuploidy [1,3]. However, in most cases spindle defects arrest the cell cycle in mitosis by activating the spindle assembly checkpoint, and lead to cell death.

Experiments in recent years suggested that the centrosome might also play a role at the transition from G1 into S-phase in the cell cycle. [4] and [5] showed that removing the centrosome from untransformed cultured cells by microsurgery or by laser ablation arrests the cell cycle in G1 phase. Further experiments by different research groups indicated that inhibition or silencing of individual centrosome components also impedes cell cycle progress into S-phase [6-11]. Molecular analysis revealed that this is due to activation of the stress signalling pathway, by activating the kinase p38 and the p53-dependent G1/S checkpoint control system [10-12].

We think that centrosome proteins may represent new original targets for anticancer therapy. Consistent with this idea, inhibiting the expression of several centrosome proteins has recently been found to sensitize lung cancer cells to the chemotherapeutic agent paclitaxel: in a synthetic lethal screen to identify genes that reduce cell viability when silenced by siRNA in the presence of sublethal concentrations of paclitaxel, several proteins of the gamma-tubulin ring complex were identified among the top targets [13]. The list of these proteins comprises gamma-tubulin, GCP2, GCP3, GCP5, and NEDD1. GCP2, 3, and 5 belong to a family of related proteins containing so-called 'grip' motifs (gamma-tubulin ring complex motifs), and together with GCP4, GCP6, and gamma-tubulin, they form the core of the gamma-tubulin ring complex (gamma-TuRC). NEDD1 has been proposed to associate peripherally with the gamma-TuRC, and to act as a recruitment factor to anchor gamma-TuRCs to the centrosome [14,15]. The function of the gamma-TuRC at the centrosome is to nucleate microtubules, supporting the assembly of the mitotic spindle. Depletion of NEDD1 inhibits gamma-TuRC recruitment to the centrosome, preventing centrosomal microtubule nucleation and the formation of a functional spindle [14,15]. Depleted HeLa cells are blocked in mitosis due to activation of the spindle assembly checkpoint [14]. Combined with the findings by [13], these results suggest that NEDD1 may represent an interesting, novel anti-cancer target. To determine whether NEDD1 constitutes a potential target for future anti-cancer therapy, we investigate here the consequences of NEDD1-depletion by RNA silencing in a variety of cancer cell lines, and we analyse the effects of depletion on the cell cycle and on potential sensitisation to anti-mitotic agents.

## Results

**Figure 1:**
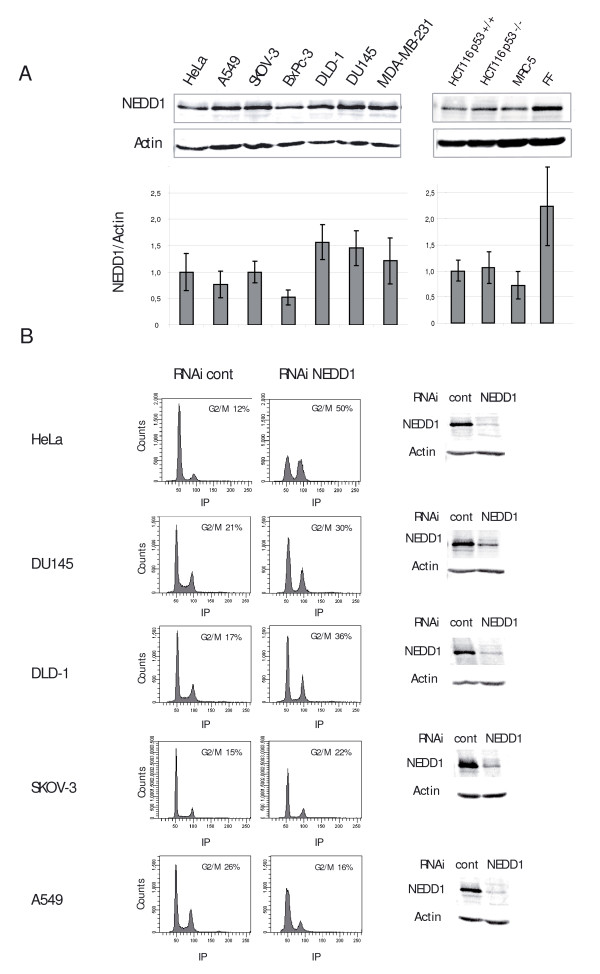
**NEDD1 expression levels in cancer cell lines and effect of depletion on the cell cycle**. (A) NEDD1 expression levels were analysed by immunoblotting using anti-NEDD1 antibody. Anti-actin antibody was used as a control. Bottom: ratio of the signals of NEDD1/actin, obtained by quantitative immunoblotting from five independent experiments (error bars represent SD). (B) Flow-cytometric analysis of cell lines treated with control siRNA or siRNA targeting NEDD1 at 50 nM for 72 h, and labelled with propidium iodide. Depletion levels of NEDD1 are shown for each cell line. Immunoblots were probed with anti-NEDD1 and anti-actin antibodies.

To assess the importance of NEDD1 for cancer cell growth, we tested a variety of cell lines for NEDD1 expression by immunoblotting. These included HeLa (cervix carcinoma), DU145 (prostate carcinoma), DLD-1 (colon carcinoma), SKOV-3 (ovarian adenocarcinoma), MDA-MB-231 (breast carcinoma), BxPc-3 (pancreas adenocarcinoma), and A549 (lung carcinoma). NEDD1 levels were normalised using actin as a loading control, and HeLa cells were used as a reference because these cells have been previously used for the characterization of the protein [14,15]. Figure 1A shows that the amounts of NEDD1 do not vary much between the cell lines. Moreover, similar amounts were detected in two different cell lines derived from colon carcinoma (HCT116 p53+/+ and -/-), and in non-cancerous cells such as MRC-5 fibroblasts and foreskin fibroblasts (Figure 1A), indicating that NEDD1 expression is not strongly deregulated in cancer cells.

In a second step, we examined the consequences of NEDD1-depletion on the cell cycle by flow cytometry. To lower the protein levels of NEDD1, we performed RNA silencing for 72 hours with previously characterized siRNA oligomers [14]. Because of different transfectability and different response to siRNA treatment, the efficiency of NEDD1-depletion varied between the different cell lines. As shown in Figure 1B, cell cultures of HeLa, DU145, DLD-1, SKOV-3, and A549 were efficiently depleted (removal of more than 70% of NEDD1). In MDA-MB-231 and BxPc-3 cells, RNA silencing was less efficient and cell cycle analysis not done. Reduced NEDD1 expression led to a significant accumulation of HeLa cells in G2/M phase (increase from 12% to 50%), consistent with the accumulation in mitosis described in previously published work [14]. An increase in G2/M levels was also noticeable, although less dramatically (but reproducibly), in DLD1, DU145 and SKOV-3 cells (Figure 1B). These cells display a higher doubling time than HeLa, which could explain why they did not efficiently accumulate in mitosis. Indeed, treatment of DU145 cells for 96 h significantly increased the relative amount of G2/M phase (51%; data not shown).

**Figure 2:**
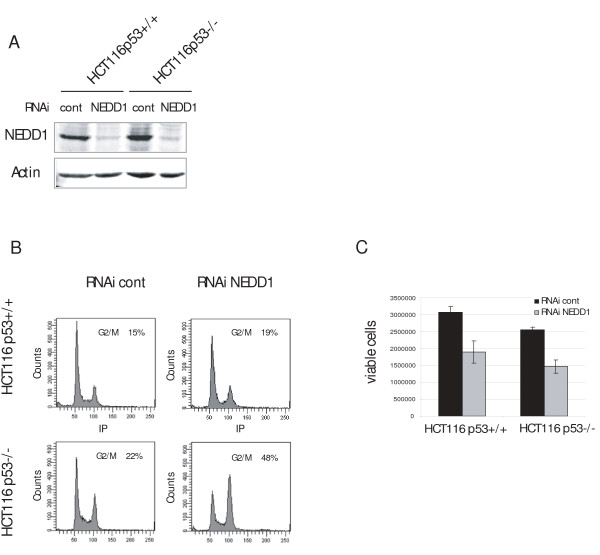
**Depletion of NEDD1 reduces cell proliferation of HCT116 p53+/+ and HCT116 p53 -/- cells**. (A) Immunoblots of HCT116 p53+/+ and HCT116 p53 -/- cells treated with control siRNA or siRNA targeting NEDD1, at 50 nM for 72 h. Blots were probed with antibodies against NEDD1 and actin. (B) Flow-cytometric analysis of HCT116 p53+/+ and HCT116 p53 -/- cells treated with control RNA or NEDD1-siRNA for 48 h, and labelled with propidium iodide (C) Number of living cells quantified by trypan blue exclusion analysis, 48 h after transfection of control RNA or NEDD1-siRNA, at 25 nM (error bars represent SD from three independent experiments).

**Figure 3:**
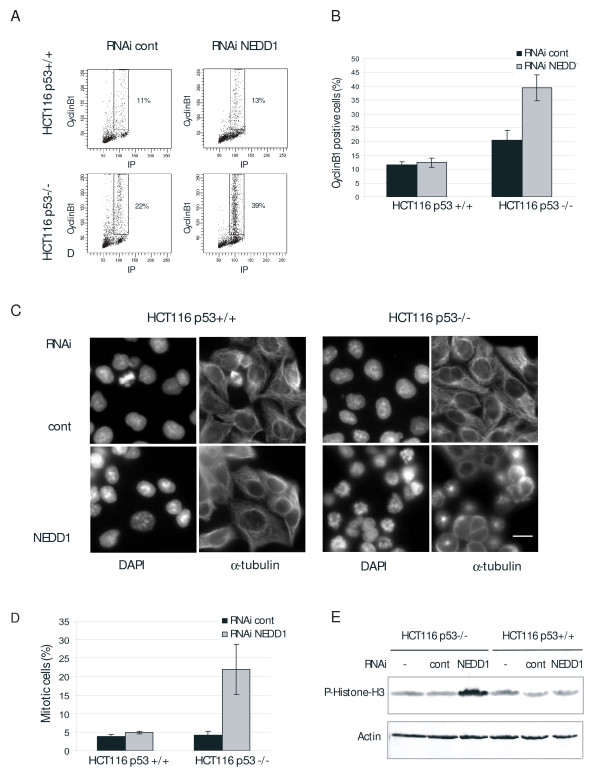
**Mitotic arrest after NEDD1-depletion depends on the checkpoint protein p53 in HCT116 cells**. (A) Flow-cytometric analysis of HCT116 p53+/+ and HCT116 p53 -/- cells treated with control RNA or siRNA targeting NEDD1 at 50 nM for 72 h and double-labelled with propidium iodide and FITC-conjugated anti-cyclinB1 antibody. (B) Quantification of cyclinB1-positive cells (error bars represent SD from three independent experiments). (C) Immunofluorescence of mitotic HCT116 p53+/+ and p53 -/- cells, 72 h after transfection with control or NEDD1-siRNA, using anti-alpha tubulin and DAPI staining. Bar, 20 μm. (D) Percentage of mitotic cells shown in (C). 500 cells were scored per condition (error bars represent SD from three independent experiments). (E) Immunoblots of HCT116 p53+/+ and p53 -/- cells treated 48 h with control or NEDD1-siRNA. Blots were probed with antibodies against phospho-histone H3 and actin.

Interestingly, A549 cells showed a decrease of the percentage in G2/M phase (from 26% to 16%). A549 cells express wild-type p53 protein, in contrast to HeLa, DLD1, DU145, and SKOV-3, in which the p53-dependent checkpoint control is compromised due to regulatory defects or mutations in p53. We therefore reasoned that different flow cytometry profiles might be due to differences in checkpoint control in the various cell lines. The observed decrease of A549 cells in G2/M phase was accompanied by an increase of the G1/S population. This is consistent with other data indicating that the G1/S transition is inhibited if centrosome integrity is disturbed in p53 wild-type cells [10-12]. To investigate this problem in more detail, we focused on the isogenic cell lines HCT116 p53 +/+ and p53 -/- (Figure 2A, Figure 2B, Figure 2C) that differ only in the expression of the p53 gene [16]. Depletion of more than 80% of NEDD1 was achieved by RNA silencing for 48 hours in both cell lines (Figure 2A). Flow cytometry clearly showed that only p53-negative cells accumulated in G2/M phase upon NEDD1-depletion (increase from 22% to 48%, Figure 2B). Consistently, the percentage of p53-negative cells with cyclin B1 staining nearly doubled (from 22% to 39%), indicating that cells accumulate in mitosis, whereas the percentage of p53-positive cells in mitosis remained approximately constant (Figure 3A, Figure 3B). To further resolve how p53-negative cells accumulate in mitosis, we performed immunofluorescence analysis of HCT116 cells by staining for microtubules. Counting of mitotic figures revealed a five-fold increase of the mitotic index in p53-negative cells, accumulating at approximately 22% in mitosis with monopolar spindles, whereas p53-positive cells didn't show any significant increase of mitotic stages (Figure 3C, Figure 3D). Consistently, p53-negative cells showed an increase of phosphorylated histone H3 by immunoblotting, indicative of mitotic accumulation (Figure 3E).

**Figure 4:**
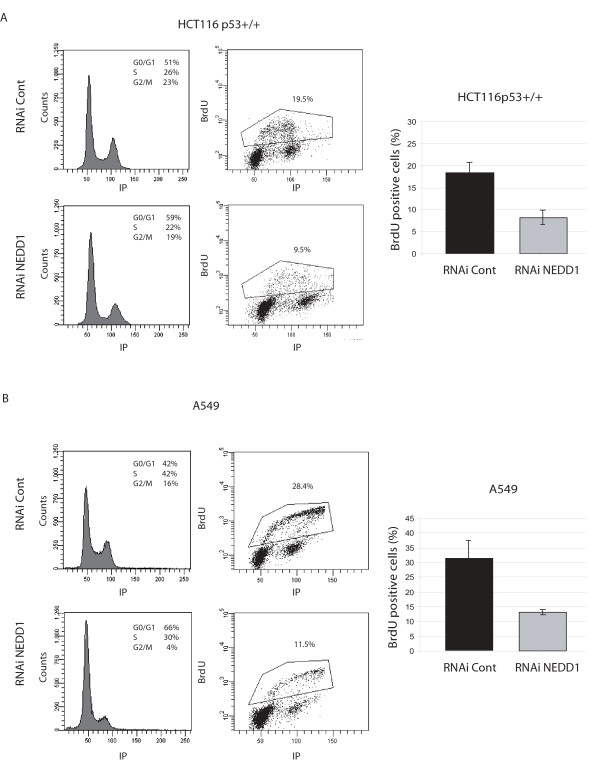
**Depletion of NEDD1 prevents DNA replication and leads to cell cycle arrest in G1 or G0 phase in HCT116 p53+/+ and A549 (p53WT) cell lines**. HCT116 p53+/+ (A) or A549 (B) cells were treated with control siRNA or siRNA targeting NEDD1 at 50 nM for 48 h, then inbubated with BrdU for 90 minutes. Left: Flow-cytometric analysis of cells labelled with 7-AAD and FITC-conjugated anti-BrdU antibody. Right: Quantification of BrdU incorporation in control or NEDD1-siRNA-treated cells. 20,000 cells were scored per condition (error bars represent SD from three independent experiments).

To examine the fate of p53-positive cells, we performed labelling experiments with bromodeoxyuridine (BrdU), which is incorporated into newly synthesized DNA in cells during S-phase (Figure 4). Our experiments show that p53-positive HCT116 cell cultures incorporate less than half of the regular BrdU amounts, indicating that cells are blocked or delayed prior to S-phase (Figure 4A, Figure 4B). Equivalent results were obtained with the A549 cells (Figure 4C, Figure 4D). Thus we show that cells respond differently to NEDD1-depletion dependent on their p53 status: in the absence of functional p53 cells arrest in mitosis, but in cells expressing wild type p53 the cycle already stops in G1/S. Despite these differences, suppression of NEDD1 reduced the proliferation of both p53-positive and negative HCT116 cells equally, by approximately 40% after 48 h siRNA treatment (Figure 2C).

**Figure 5:**
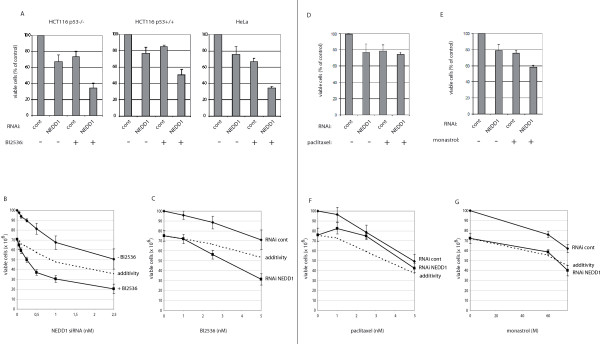
**The Plk1 inhibitor BI2536 and siRNA against NEDD1 act synergistically to reduce cell viability**. (A) Quantification of HCT116 p53+/+, p53-/-, or HeLa cells surviving after control RNA or NEDD1-siRNA transfection (0.5 nM) alone, or combined with BI2536 (10 nM in HCT116 cells, 5 nM in HeLa cells). The percentage of surviving cells is indicated as a percentage of the number of control cells (treated with control siRNA, without BI2536), quantified by trypan blue exclusion analysis after 48 h incubation. (B) Percentage of viable HeLa cells after 48 h treatment with variable concentrations of NEDD1-siRNA alone, or combined with 5 nM BI2536. (C) Percentage of viable HeLa cells after 48 h treatment with variable concentrations of BI2536 combined with 0.5 nM control or NEDD1-siRNA. (D, E) Percentage of viable HeLa cells after 48 h treatment with control or NEDD1-siRNA (0.5 nM) alone, or combined with paclitaxel (2.5 nM, D) or monastrol (60 μM, E). (F, G) Percentage of viable HeLa cells after 48 h treatment with variable concentrations of paclitaxel (F) or monastrol (G) combined with 0.5 nM control or NEDD1-siRNA. Error bars represent SD from six (A) or three (B-G) independent experiments. In B, C, F, G, dose-response curves for theoretical additive effects, calculated according to [22], are included for comparison (dotted line).

Because depletion of proteins of the gamma-tubulin complex sensitizes p53-mutated cells derived from non-small cell lung cancer for the treatment with the chemotherapeutic agent paclitaxel [13], we wanted to confirm that specific depletion of NEDD1 had a similar effect in HeLa and in HCT116 cells with and without p53 (Figure 5A, Figure 5B, Figure 5C, Figure 5D, Figure 5E, Figure 5F, Figure 5G). The previously published screen for chemosensitizers by [13] was performed at high concentrations of siRNA for 48 h, followed by 48 h treatment with paclitaxel. When we applied high doses of siRNA against NEDD1 in our own experiments for 48 h, we already noticed a high cytotoxicity before paclitaxel addition (Figure 2C and data not shown). We thus lowered the concentration of siRNA and added paclitaxel 4 hours after the start of the transfection, for a total of 48 h of transfection. Cells were treated with a range of low concentrations of siRNA (0–2.5 nM) and of paclitaxel (0–5 nM). However, under these conditions, no significant sensitising effect was observed when both treatments were combined (Figure 5D, Figure 5F, and data not shown). In Figure 5D, HeLa cells were treated with 0.5 nM NEDD1-siRNA and 2.5 nM paclitaxel. Alone, these treatments yielded approximately 80% of viable cells compared to cells treated with control siRNA. Simultaneous application of both paclitaxel and NEDD1-siRNA did not reduce further the number of viable cells. The same results were found at variable doses of paclitaxel (Figure 5F). Subsequently, we tested other anti-mitotic agents: BI2536 is a potent and specific inhibitor of the mitotic kinase Plk1 and inhibits tumor growth in vivo [17]. Both, the inhibition of Plk1 and the depletion of NEDD1 produce similar phenotypes: inhibition of gamma-TuRC recruitment to the centrosome and inhibition of centrosomal microtubule nucleation, yielding monopolar spindles in mitosis [14,15,18-20]. We therefore tested whether siRNA targeting NEDD1 would sensitise cells to the Plk1 inhibitor. HeLa and HCT116 cells (p53 +/+ and -/-) were treated with 0.5 nM siRNA and 5 nM (HeLa) or 10 nM (HCT116) BI2536 for 48 h (Figure 5A). In all three cell lines, combined treatment reduced the number of viable cells further than any independent treatment: in HCT116 p53-/- cells, combined treatment yielded 34% of viable cells compared to cells treated with control siRNA, whereas single NEDD1-siRNA or drug treatment yielded 67% or 74% of viable cells respectively. In HCT116 p53+/+ cells, 50% of viable cells were counted after combined treatment, compared to 77% after siRNA or 85% after drug treatment alone. In HeLa cells, 33% of viable cells were counted after combined treatment, compared to 77% or 66% after single siRNA or drug treatment, respectively. Calculation of the combination index (CI), based on the dose response curves of single NEDD1-siRNA and BI2536 treatment (Figure 5B, Figure 5C, and data not shown) led to a value of CI < 0.3, indicating a strong synergy [21]. For comparison, combination of NEDD1-siRNA and taxol led to a value of CI > 1 which indicates antagonism. A more detailed analysis was performed in HeLa cells, treated with a range of low concentrations of siRNA. The graph in Figure 5B shows dose response curves after treatment with NEDD1-siRNA, alone or in combination with the Plk1 inhibitor, as well as a theoretical additive effect, calculated according to [22] (see 'Methods' for details). The graph illustrates that synergy occurs even at the lowest concentrations of NEDD1-siRNA. Inversely, treatment with NEDD1-siRNA also increased the anti-proliferative effect of BI2536 at a range of concentrations below 5 nM of this drug (Figure 5C).

Finally, we tested a possible synergy with monastrol, a specific inhibitor of Eg5, which also blocks cells in mitosis by inducing monopolar spindles [23]. HeLa cells were treated as described for paclitaxel and BI2536. Figure 5E shows an enhancement of the anti-proliferative effect following combined treatment with 0.5 nM NEDD1-siRNA and 60 μM monastrol, yielding 58% of viable cells compared to 79% or 76% after single siRNA or drug treatment, respectively. However, in this case calculation of the combination index indicated an additive effect (CI = 1), but no synergy. This effect was also seen at higher doses of the drug (Figure 5G).

To better understand the mechanism of the synergy between siRNA against NEDD1 and Plk1 inhibition, we analysed the cell cycle profiles by flow cytometry following treatments.

**Figure 6:**
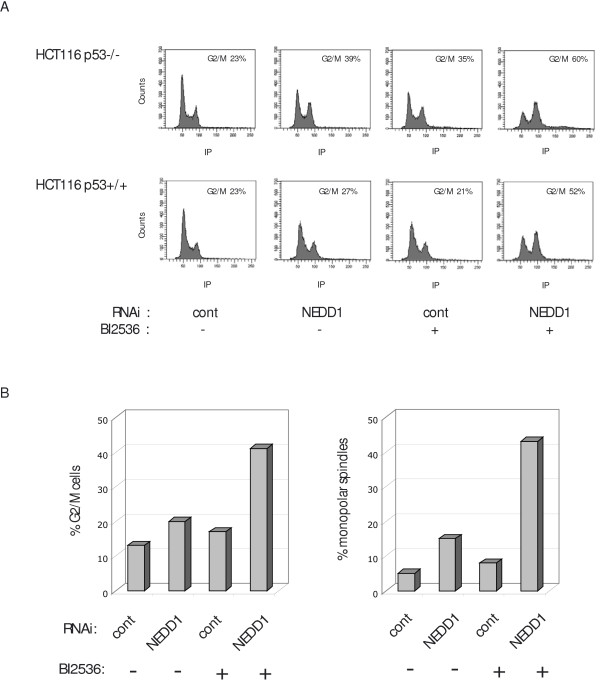
**Depletion of NEDD1 potentiates the anti-mitotic activity of the Plk1 inhibitor BI2536**. (A) Flow-cytometric analysis of HCT116 p53+/+ and p53-/- cell lines treated for 48 h with control or NEDD1-siRNA (0.5 nM) alone or combined with BI2536 (10 nM) and labelled with propidium iodide. (B) Quantification of G2/M HeLa cells following flow-cytometric analysis, after treatment for 48 h with control or NEDD1-siRNA (0.5 nM) alone, or combined with BI2536 (5 nM). (C) Quantification of monopolar spindles (as a percentage of total prometa-metaphase spindles) present in HeLa cells treated for 48 h with control or NEDD1-siRNA (0.5 nM) alone, or combined with BI2536 (5 nM).

Treatment of HCT116 cells with either NEDD1-siRNA or BI2536 alone showed little effect on the cell cycle compared to control siRNA treatment (Figure 6). Only a subtle increase in the G2/M population was seen, whereas simultaneous treatment led to a strong enhancement of the G2/M peak (Figure 6A). Remarkably, this was found in both p53 +/+ and -/- cells. Similar results were found in HeLa cells (Figure 6B). Moreover, analysis of the mitotic figures by immunofluorescence revealed that the increase of the G2/M population correlated with larger amounts of monopolar spindles (Figure 6B). Thus combination of both treatments enhances their anti-mitotic effect due to an enhanced inhibition of bipolar spindle formation.

## Discussion

Cells in mitosis represent a validated target in anti-cancer therapy. Strategies that are currently used, such as treatment with microtubule inhibitors, e.g. taxans or vinca alkaloids, arrest cycling cells in mitosis by interfering with microtubule dynamics. Because of secondary effects in normal cells and also because of resistance of cancer cells to such treatments, new targets have been explored, leading to the development of inhibitors of mitotic kinases such as Plk1 or Aurora, or of motor proteins such as Eg5 [24]. These proteins control the transformation of the microtubule network into a functional bipolar spindle. Inhibition of these factors induces abnormal spindles, followed by mitotic arrest due to activation of the spindle assembly checkpoint, resulting in frequent cell death. Likewise, the centrosome protein NEDD1 is essential for spindle assembly. More specifically, NEDD1 is needed for the sudden increase of gamma-TuRC-binding to the centrosome at the onset of mitosis, and for the resulting increase of microtubule nucleation [14,15,25]. We have previously shown that depletion of NEDD1 from HeLa cells induces accumulation of cells in mitosis with monopolar spindles [14]. We show here that NEDD1 expression is not deregulated in cancer cells: NEDD1 is expressed at comparable levels in a variety of cancer cell lines and in normal cells. However, lowering its expression slows down cell proliferation and enhances the anti-proliferative effect of Plk1 inhibition.

Treatment with siRNA against NEDD1 has different effects on the cell cycle depending on the p53 status of the cell lines: accumulation of cells in G1 is observed if the p53-dependent G1/S checkpoint is functional, consistent with published data indicating that centrosome abnormalities can trigger the cellular stress response via p38 and cause p53-dependent cell cycle arrest [10-12]. In cells without a functional p53-dependent checkpoint, the cell cycle is arrested during mitosis, due to the formation of monopolar spindles. Moreover, we show that very low concentrations of NEDD1-siRNA treatment potentiate the mitotic inhibition induced by BI2536, a strong inhibitor of Plk1 [17,18]. Remarkably, this effect is independent of the p53 status of the cells. Under the conditions of our experiment, using low concentrations of siRNA, the treatment did not induce a significant G1/S arrest in HCT116 p53+/+ cells, probably because the stress response was not triggered [12]. Thus, cells passed the G1/S checkpoint and arrested in mitosis due to the combined effect of the reduction of NEDD1 levels and Plk1 inhibition.

We demonstrate that siRNA against NEDD1 and inhibition of Plk1 act synergistically to reduce cell numbers, exceeding by far a simple additive effect of both treatments. We show that the reduced viability after simultaneous treatment is associated with an increase of abnormal mitotic spindle assembly. Individual treatments produce similar spindle defects in mitosis: NEDD1 or Plk1-dependent defects induce formation of monopolar spindles which activate the spindle assembly checkpoint [14,15,18-20,26-31]. We think that Plk1 and NEDD1 act in a common pathway: they are both necessary for the recruitment of the gamma-TuRC to the centrosome early in mitosis [14,15,18,28,31]. This is a prerequisite for amplification of microtubule assembly and for the separation of the spindle poles. Thus inhibition of both proteins would amplify the loss of gamma-TuRC recruitment and the formation of monopolar spindles, producing a synergistic effect.

In contrast, monastrol also produces monopolar spindles and activates the mitotic checkpoint, but only yields an additive effect together with NEDD1 depletion. Likely, this is because monastrol acts in a different, parallel pathway of spindle assembly, by inhibiting the motor protein Eg5 and disrupting the forces of antiparallel microtubule sliding that is necessary to separate the two centrosomes [23].

At low concentration of paclitaxel, we see neither a synergistic, nor an additive effect on the proliferation of cells when combined with siRNA against NEDD1. At first glance, these results seem to contrast with the screening data of [13]. However, our experiments were performed in different cell lines, at lower concentration of siRNA against NEDD1, and using different durations of treatment. We think that the discrepancies might be explained as follows: first, under the conditions of [13], cells are depleted of NEDD1 with high doses of siRNA before paclitaxel treatment, which should arrest them in mitosis before application of the drug. In these mitotic cells, paclitaxel may have an enhanced cytotoxicity as compared to interphase cells. Second, it is known that sensitivity to paclitaxel varies considerably between cancer cell lines, with more than 20-fold variation of the apoptotic index [32]. Combination treatment might thus yield different results depending on the cell line.

## Conclusion

Altogether, our data suggest that interfering with the function of NEDD1 slows down proliferation, in particular in combination with other reagents that block mitosis, such as Plk1 inhibitors. Suppression of NEDD1 induces defects in centrosome function that arrest the cell cycle. Whether NEDD1 is indeed a potential target for cancer therapy remains to be determined in animal models in vivo. At present, it is encouraging to note that even very low doses of siRNA against NEDD1 affect cell growth in synergy with Plk1 inhibition. Moreover, under these conditions we observe no apparent abnormalities on interphase microtubules (unpublished observation). Therefore, the combination of inhibitors against NEDD1 and other mitotic targets should have the advantage for eventual therapeutic use to produce minimal side effects in interphase cells and should thus lower the toxicity on non-cancerous cells. Targeting centrosomal proteins involved in microtubule assembly, such as NEDD1, represents a novel, challenging area of research for new anti-mitotic compounds.

## Methods

### Cell lines

Human cervix adenocarcinoma, HeLa (ATCC CCL-2), human prostate carcinoma DU145 (ATCC HTB-81), human colon adenocarcinoma DLD-1 (ATCC CCL-221), ovarian adenocarcinoma SKOV-3, human breast adenocarcinoma MDA-MB-231 (HTB-26), human pancreas adenocarcinoma BxPc-3 (ATCC CRL-1687), human lung carcinoma A549 (ATCC CCL-185) and normal human lung fibroblast MRC-5 (ATCC CCL-171) cell lines were purchased from American Type Culture Collection (Manassas, VA). HeLa and MRC-5 cells were maintained in Dulbecco's Modified Eagle's Medium supplemented with 2 mM L-GlutaMAX I and 10% fetal bovine serum (FBS). DLD-1 and DU145 were grown in Minimum Essential Medium supplemented with 2 mM L-Glutamine and 10% FBS. SKOV-3 and BxPc-3 were cultured in RPMI 1640 medium without phenol red, supplemented with 2 mM L-Glutamine and 10% FBS. A549 and MDA-MB-231 were maintained in RPMI 1640 medium supplemented with 2 mM L-Glutamine and 10% FBS. The human colon cancer cell lines HCT116 p53 +/+ and HCT116 p53 -/- [16], kindly provided by Dr. Bert Vogelstein (Johns Hopkins University School of Medicine, Baltimore, MD, USA), were grown in McCoy's 5A medium with 10% FBS. Primary normal foreskin fibroblasts were from Dr. A. Popov and Dr. A. Juhem (INSERM U 836, Grenoble Institute of Neurosciences) and were grown in Dulbecco's Modified Eagle's Medium supplemented with 2 mM L-GlutaMAX I and 10% FBS.

### RNA silencing

Double-stranded siRNA oligomers, targeting nucleotides 229–247 of human NEDD1 (GGGCAAAAGCAGACAUGUG) were used [14]. Control (non-silencing) siRNA was provided by QIAGEN. Cells were plated to obtain a confluency of 70 to 90% at 48 to 72 hours after transfection. Transfections were carried out using Lipofectamine RNAiMax (Invitrogen), according to the manufacturer's protocol. High concentrations of siRNA were used in Figure 1, Figure 2, Figure 3, and 4: 50 nM, and in figure. 2D: 25 nM. Low concentrations were used in Figure 5 and Figure 6: 0.5 nM, or concentrations varying from 0–2.5 nM.

### Flow cytometry

Cells treated with siRNAs for 48 or 72 h were harvested with trypsin-EDTA, washed twice with PBS and stained with propidium iodide using a Coulter DNA Prep Reagents kit (Beckman-Coulter), according to the manufacturer's instructions. Analysis of the DNA content was done using a LSRII flow cytometer (Becton-Dickinson). For each experiment, 20,000 cells were analyzed using a 'doublet discrimination model' to exclude aggregates of multiple cells from the analysis, and data were processed using Diva software (Becton-Dickinson). The percentages of cells at G2/M phase of the cycle were calculated using ModFit LT Software (Becton-Dickinson). Cell cycle analysis on the basis of cyclin B1 immunofluorescence was performed in 10,000 cells/experiment, at 72 hours after siRNA transfection. Cells were rinsed in ice-cold PBS after trypsinization, and fixed in 75% ice-cold ethanol overnight at -20°C. Samples were then rinsed once with PBS containing 1% FBS, and lysed for 5 min at 4°C with 0.25% Triton X-100 in the same buffer. After two further rinses, cells were incubated for one hour at room temperature with 10 μl of fluorescein-conjugated mouse anti-human cyclin B1 antibody (GNS-1, BD Pharmingen), or with fluorescein-conjugated mouse IgG1 monoclonal isotype control antibody (MOPC-21, BD Pharmingen). After rinsing twice with PBS containing 1% FBS, cells were incubated for 45 minutes at 4°C in 1 ml PBS containing 10 μg/ml propidium iodide (Fluka).

The rate of DNA synthesis was examined by a BrdU incorporation method using a FITC BrdU Flow Kit (BD Pharmingen) according to the manufacturer's instructions. Briefly, 48 h after siRNA transfection cells were incubated for 1 hour at 37°C with 32.5 μM BrdU. Cells were then harvested, permeabilized, and stained with a FITC-labeled antibody against BrdU followed by the addition of 7-amino-actinomycin D (7-AAD). Cell cycle analysis was performed as described above using LSRII flow cytometer and Diva Software (Becton-Dickinson). Cultured cells without BrdU were used as nonspecific binding controls for the FITC-labeled anti-BrdU mAb.

### Western blot analysis

Cells were harvested with trypsin-EDTA, washed twice with PBS then lysed with ice-cold lysis buffer (50 mM Tris, pH 7.5, 150 mM NaCl, 1% Triton-X100, 2 mM EDTA) supplemented with protease inhibitor cocktail (Complete, EDTA-free, Roche) and 1 mM DTT. After sonication, 50 μg of total protein were separated on SDS-PAGE and electrotransferred onto nitrocellulose membranes. Membranes were incubated for 2 h with antibodies against NEDD1 at 1:5,000 dilution [14], phospho-histone-H3 at 1:500 dilution (rabbit polyclonal anti-phospho-Histone H3 Ser10 antibody, Upstate), or actin at 1:10,000 dilution (mouse monoclonal anti-actin MAB1501), in blocking buffer (Li-cor Biosciences) containing 0.1% Tween-20. As secondary antibodies, IRDye 800CW-conjugated goat anti-rabbit IgG (Li-cor) and IRDye 680CW-conjugated goat anti-mouse IgG were used for 1 h at 1:10,000 dilution in blocking buffer (Li-cor), containing 0.1% Tween-20 and 0.01% SDS. The protein levels of NEDD1, phospho-histone-H3 and actin were determined with the Odyssey imaging system (Li-cor Biosciences).

### Immunofluorescence analysis

72 h after siRNA transfection, cells grown on coverslips were fixed in methanol at -20°C for 15 minutes. After rinsing three times with PBS, cells were incubated with mouse monoclonal anti-alpha tubulin antibody (Sigma-Aldrich, 1:1,000) in PBS + 0.1% Tween-20 + 1% BSA, for 1 h at room temperature. Following three rinses with PBS, Alexa 488 goat anti-mouse antibody (Molecular Probes, 1:1,000 in PBS + 0.1% Tween-20 + 1% BSA) was added for 45 minutes. DNA was labeled with DAPI (1 μg/ml). Cells were examined with a fluorescence microscope (Axiovert, Carl Zeiss MicroImaging) using a 63x/1.4NA Plan-Apochromat lens. Images were acquired with an AxioCam MRm camera and AxioVision software (Carl Zeiss MicroImaging). Mitotic indices were determined using morphological criteria, by counting cells that display DNA condensation and mitotic spindles.

### Drug treatment and combination analysis

2 × 10^5 ^cells were plated in 6-well plates (35 mm in diameter) and allowed to adhere for 24 hours; the transfection of double-stranded siRNA oligonucleotides was done as described above, at variable concentrations (0–2.5 nM). For combination studies, cells were transfected first and BI2536 (kindly provided by the 'Centre de Recherche en Oncologie Pierre Fabre'), paclitaxel or monastrol (Sigma) were added 4 hours after transfection in fresh medium. Cells were harvested 48 hours after the beginning of transfection, and viable cells were counted by trypan blue exclusion analysis using a Vi-CELL Viability Analyzer (Beckman Coulter).

In Figure 5A, Figure 5D, Figure 5E, the combination indices (CI) were calculated using the following equation [21]:

CI = C_N,x_/IC_x,N _+ C_D,x_/IC_x,D_, where C_N,x _and C_D,x _are the concentrations of NEDD1-siRNA and drugs (BI2536, paclitaxel or monastrol) used in combination to achieve x% drug effect, and IC_x,N _and IC_x,D _are the concentrations for single agents to achieve the same effect. A CI of less than 1 indicates synergy, equal to 1 indicates additivity, and more than 1 indicates antagonism [21]. In Figure 5B, Figure 5C, Figure 5F, Figure 5G, the theoretical dose-response curves for additivity were calculated using the formula [22]:

TA = E_N _- (E_D _× E_N_/100) (Figure 5B) or TA = E_D _- (E_D _× E_N_/100) (Figure 5C, Figure 5F, Figure 5G), where E_N _is the effect of NEDD1-siRNA alone, and E_D _is the effect of the drug alone in %.

## Abbreviations

BrdU: 5-bromo-2-deoxyuridine; DAPI: 4',6-diamidino-2-phenylindole; Eg5: egg protein 5; GCP: gamma-tubulin complex protein; NEDD1: neural precursor cell expressed, developmentally down-regulated gene 1; Plk1: polo-like kinase 1; CI: combination index; SD: standard deviation.

## Competing interests

The authors declare that they have no competing interests.

## Authors' contributions

V.T. and L.H. contributed equally, by performing cell culture experiments, transfection, immunofluorescence, western blotting, data analysis, and participating in drafting the manuscript. N.R. carried out flow cytometry and data analysis. C.E. participated in the design and analysis of the pharmacological experiments, and in drafting the manuscript. A.M. participated in the conception of the project, coordinated the study, and participated in drafting the manuscript. All authors read and approved the final manuscript.
